# Efficacy and Tolerability of Hybrid Complexes of High- and Low-Molecular-Weight Hyaluronan Intradermal Injections for the Treatment of Skin Roughness and Laxity of the Neck

**DOI:** 10.1155/2022/4497176

**Published:** 2022-09-17

**Authors:** Adele Sparavigna, Laura Bombelli, Andrea Maria Giori, Gilberto Bellia

**Affiliations:** ^1^DERMING S.r.l., Milano, Italy; ^2^IBSA Farmaceutici Italia, Lodi, Italy

## Abstract

This study aimed to evaluate the efficacy of a well-characterized formulation of hyaluronic acid (HA), Profhilo®, in the treatment of roughness and laxity of the neck skin. The study was performed on 25 subjects ranging in age from 40 to 65 years. Two injections of the studied product at 30-day intervals were performed, with evaluations conducted 1 and 4 months after the first injection. The efficacy was determined by clinical and multilevel instrumental evaluations. In addition, at the end of the study, the subjects completed a questionnaire related to the efficacy and tolerability of the product. The studied product was shown to induce a clear and statistically significant improvement in the skin of the neck in all the subjects, with concordant results between the clinical, instrumental, and subjects' evaluations. The positive effects, present after the first injection, were further increased in the second and last evaluation. Notably, the product was reported to have a very high tolerability by both clinicians and subjects. In conclusion, two injections of the studied product safely induced skin amelioration in subjects with mild to moderate neck skin roughness and laxity.

## 1. Introduction

The field of aesthetics is continuously expanding due to the high demand for amelioration in ageing people. The neck in particular is of interest, being an area in which with age there are several, often undesired, changes requiring interventions. The neck, together with the face and sometimes arms, is a part of the body visible to other people, and in contrast to the structure of the neck in youth, visible changes in skin appearance and wrinkle formation are evident in a significant proportion of subjects with increasing age [1, 2]. Several approaches have been described with the aim of improving neck appearance [3–6]. Injections of hyaluronic acid (HA) are a widely used method to restore skin appearance, and several reports have not only shown its efficacy but also it is very good tolerability [7–17]. HA can be present in different forms and with different molecular weights [7, 17–20], making it versatile for different applications. In addition, the fact that our body contains HA strongly reduces the chance of undesired allergic reactions. A particular formulation containing hybrid cooperative complexes of low- and high-molecular-weight HA has been shown to be effective and well tolerated in the treatment of facial and inner arm skin laxity [19, 21, 22]. Studies performed on fibroblasts or keratinocytes in vitro have shown that this particular formulation is superior to both low-molecular-weight HA (L-HA) and high-molecular-weight HA (H-HA) in reducing hyaluronidase-mediated degradation, increasing the release of elastin, and increasing the amount of collagens [19]. Altogether, these properties fit well with the observed increased skin elasticity induced by Profhilo® in different clinical studies [21, 22]. Moreover, the HA complex was also superior to H-HA or L-HA in inducing adipocyte differentiation in vitro and activating adipose-derived stem cells [20]. This would suggest that this HA complex is more effective in reducing fat resorption, which is associated with ageing, as well as in contributing to soft tissue augmentation and reconstruction. In the present work, we describe the efficacy and tolerability of this product for the treatment of skin laxity of the neck in female subjects.

## 2. Materials and Methods

This open, single-centre, clinical trial was conducted on 25 female subjects who gave their informed consent to participate. An expert dermatologist supervised all the procedures performed in the trial. The study was approved by an independent ethics committee and was performed according to the Helsinki Declaration relative to the ethical principles for medical research involving human subjects. The trial has been registered on clinicaltrials.gov (NCT04002856). The protocol of the study included two intradermal implants at a 1 month distance and three visits:the first visit at baseline (T0), immediately followed by the 1st injection; the second visit 1 month after the 1st injection and just before the second injection (T1), and the last one 4 months after the first injection (i.e., 3 months after the second injection [T2]). The 4-month end point was selected on the basis of the results previously obtained with the product for the treatment of face wrinkles [22].

The product injected was Profhilo® (IBSA Farmaceutici Italia S.r.l.), consisting of a prefilled syringe containing 2 ml of 3.2% HA (32 mg H-HA and 32 mg L-HA in 2 ml of saline).

**Figure 1:**
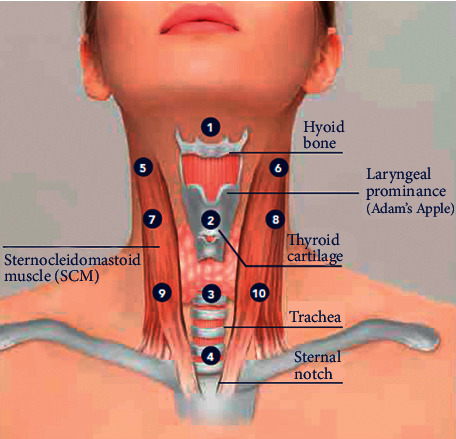
Schematic representation of the 10 injection points used in this study.

The studied product was injected into the middle-deep dermis using a 29 G needle and a bolus technique named BAP Neck (Bio Aesthetic Point Neck) consisting of a series of 10 microwheals (0.2 ml each) on 3 vertical lines, as depicted in Figure 1. The use of this technique minimizes the variation between subjects and ensures that the injection point does not touch vital structures.

**Figure 2:**
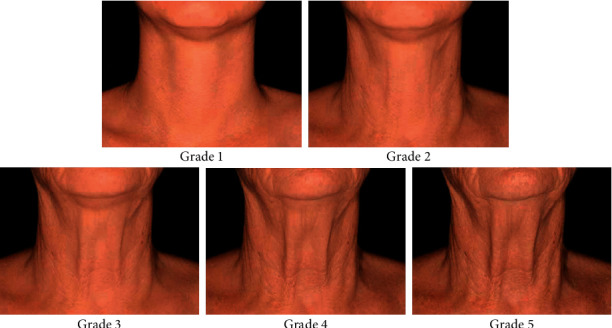
IBSA Neck laxity scale.

To be enrolled in the study, the subjects had to be female, ranging in age between 40 and 65 years, presenting a grade of 3–4 on the IBSA Neck Laxity Scale [23] and requesting restoration. This visual scale (Figure 2) is based on 5 levels from 1 (no laxity) to 5 (very severe laxity). The subjects had to ensure their availability for postprocedural follow-ups and not change their habits with respect to food, physical activity, cosmetics, and cleansing products for the neck. In addition, for the entire period, they had to avoid UV irradiation to the neck without appropriate sun protection.

Women who were pregnant or lactating, smokers, or alcohol or drug abusers were excluded from the study. Females not in menopause were excluded if they did not use contraceptive precautions or did not accept the performance of pregnancy tests at both T0 and T1 (i.e., before and 1 month after the first injection). A body mass index change (±1) during the study was considered an exclusion criterion. Additional exclusion criteria were skin treatments in the areas under study in the last 6 months before enrolment or past surgical procedures for aesthetic purposes in the same areas; known hypersensitivity to the tested product and/or its ingredients; the presence of dermatological diseases, such as dermatitis, lesions, scars, or malformation in the tested area; recurrent facial/labial herpes; and presence of active eczema, psoriasis, severe rosacea, scleroderma, infections, and severe acne in the tested area. Systemic diseases, such as diabetes; endocrine disease; hepatic, renal, or cardiac disorders; pulmonary disease; cancer; neurological disease; inflammatory or immunosuppressive disease; and allergy to drugs constituted exclusion criteria. Finally, subjects receiving concomitant treatments with anticoagulants, antiplatelets, antihistamines, corticosteroids, narcotics, antidepressants, and immunosuppressive drugs were excluded from the study. The use of drugs not previously mentioned was evaluated and judged by the investigator as a potential exclusion criterion.

### 2.1. Evaluation of the Efficacy

The efficacy of the treatment was determined using both qualitative (clinical) and quantitative (instrumental) assessments supported by photographic documentation.

Clinical neck evaluation was performed at each visit using IBSA Neck Laxity Scale scores ranging from 1 (no roughness and laxity) to 5 (very severe roughness and laxity) (Figure 2).

The photographic record was standardized among subjects in terms of the magnification factor, the intensity of the lamps, angles of incidence, and inclination of illumination.

Instrumental evaluation of the efficacy was performed using different noninvasive techniques as follows:

1. Determination of superficial skin hydration assessed by the electrical capacitance of the skin using a Corneometer CM825 (Courage-Khazaka, Koln, Germany). Three measurements on the same skin area were performed, and a mean value was calculated accordingly.
2. Determination of deep skin hydration assessed by tissue dielectric constant of superficial and deep skin layers measurements using a MoistureMeterD. This instrument generates high frequency, low power electromagnetic waves, and measures changes in the total water content of the tissue. The use of probes of different sizes allows determination at different depths. In this study, 0.5 and 1.5 mm probes were used.
3. Determination of skin plastoelasticity using torsiometry. Skin torsion was measured using a Dermal Torque Meter (Dia-Stron Ltd, UK), which measures the torsion angles during (torque on) and after (torque off) mechanical stimulus application. Different parameters can be obtained to define the immediate and maximum elasticity (Ue and Uf, respectively), viscoelasticity (Uv), and immediate elastic recovery (Ur);
4. Determination of skin density through profilometry. This evaluation was performed by pinching a small skin area of the neck of approximately 7 cm^2^ using a special mask that ensured measurements in the same area at each visit. The pinch induces changes in the skin profile, which in turn depends on the cutaneous density. The profile is then recorded using a Primos portable device (GFMesstechnick) coupled with software that compares the different images obtained.

### 2.2. Evaluation of Safety

Tolerability of the product was evaluated considering the local reactions (tardive swelling, pain, erythema, or bruising) as well as any other adverse event or reaction occurring locally or systemically.

### 2.3. Volunteer Self-Assessment

At the end of the study, the subjects were asked to complete a questionnaire in which they evaluated the product's efficacy against skin roughness and laxity of the neck with scores of very marked, marked, medium, light, and absent. Through the questionnaire, they were also able to evaluate tolerance using scores of bad, poor, good, and excellent.

### 2.4. Statistical Analysis

For clinical data, the Friedman test was followed, in cases with significant results, by the Holm–Sidak adjusted Wilcoxon signed rank test.

For the instrumental data, when the normality hypothesis was rejected by the Shapiro–Wilk normality test (5% threshold), a nonparametric test (Friedman) was used. When the normality hypothesis was confirmed, the parametric ANOVA test was used. In both cases, the Holm–Sidak adjusted test was employed in cases with significant results.

## 3. Results

Two out of 25 subjects enrolled in the study did not complete the trial due to problems not related to the study. The data were therefore collected and analysed for 23 subjects who completed the trial. The mean age was 54 (range: 41–65) years. None of the subjects had previous intradermal implants in the neck.

### 3.1. Clinical Evaluation of the Efficacy

**Figure 3:**
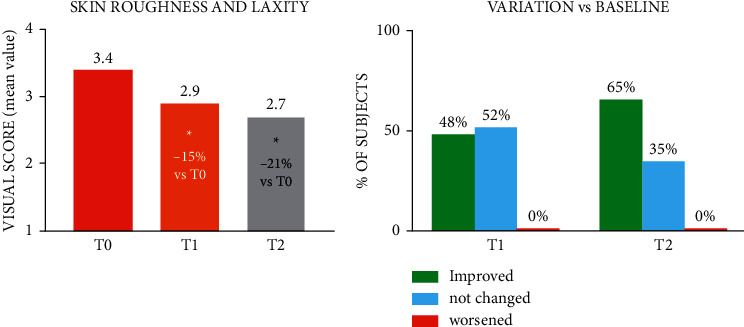
Visual score (a) and clinical score change relative to baseline (b) for skin neck roughness and laxity. ^*∗*^*p* < 0.05 vs. T0 (Holm-Sidak adjusted Wilcoxon signed rank test).

The treatment with the studied product induced a statistically significant improvement in neck skin laxity, which was already detectable at T1 (15%) and further increased at T2 (21%) (Figure 3). This improvement was associated with a reduction in the IBSA Neck Laxity Scale of at least 1 grade in more than half of the subjects.

**Figure 4:**
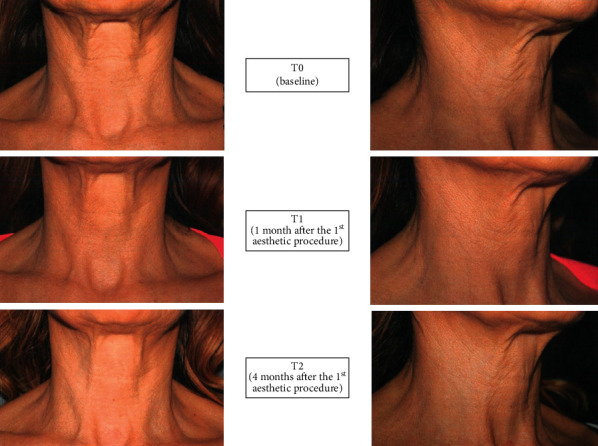
Representative photographic documentation obtained at baseline, T1 and T2.

These positive results were confirmed by photographic documentation, which clearly showed the ameliorations due to the treatment (see Figure 4 for representative images).

### 3.2. Instrumental Evaluation of the Efficacy

**Figure 5:**
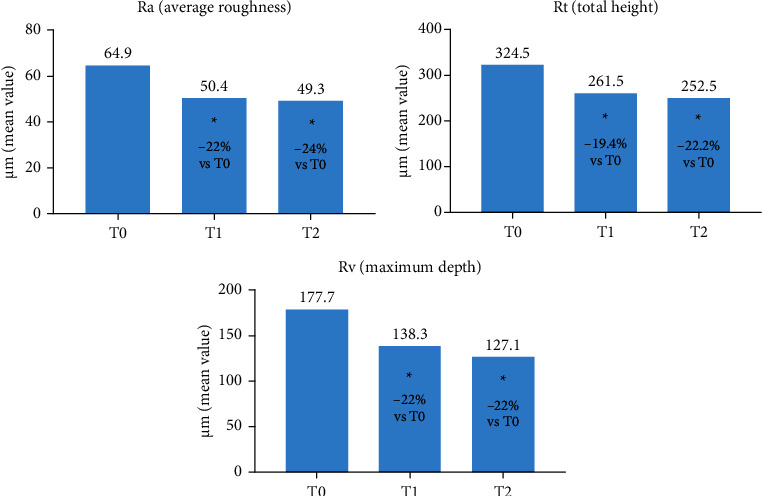
Skin density was determined through profilometric parameters. Average roughness (a), total height (b) and maximum depth (c) were determined at T0, T1 and T2. The percentage of reduction at T1 and T2 vs. T0 is reported in each bar ^*∗*^*p* < 0.05 vs. T0 (Holm-Sidak adjusted t test).

Using the Primos portable system, three different profilometric parameters (Ra, average roughness, Rt, total height, and Rv, maximum depth of analysed skin profile) defining skin density were recorded. The neck area investigated was the same for each subject and at each visit. A clear and statistically significant reduction in all three profilometric parameters was obtained at the first visit (T1) and ameliorated at the subsequent visit (T2) (Figure 5). This reduction is indicative of reduced wrinkled and sagging skin.

**Figure 6:**
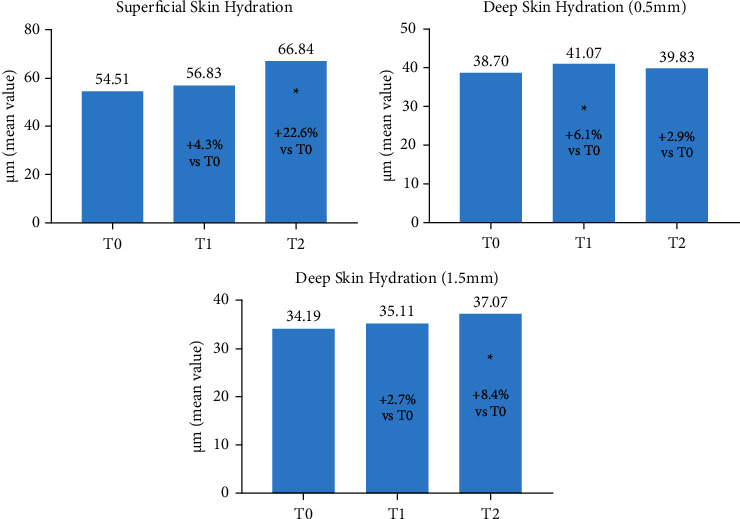
Superficial (a) and deep ((b), 0.5 mm, (c) 1.5 mm) skin layer hydration was determined at T0, T1 and T2. The percent increase at T1 and T2 vs. T0 is reported in each bar ^*∗*^*p* < 0.05 vs. T0 (Holm-Sidak adjusted t test).

The effect of treatment on skin hydration was determined by measuring the superficial and deep hydration. The latter was determined at 0.5 mm and 1.5 mm depths. With both measurements, a clear moisturizing effect was obtained both at T1 and T2, with variation from baseline ranging from approximately 3 to 22%. The graphs relative to the three evaluations are reported in Figure 6.

**Figure 7:**
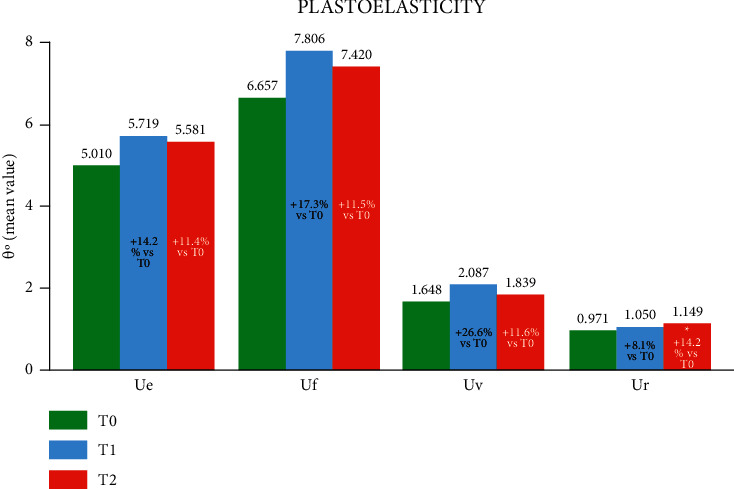
Plastoelasticity in the neck determined at T0, T1 and T2. The four parameters (Ue, Uf, Uv and Ur) determined with this technique are shown. The variations at T1 and T2 vs. T0 are reported in each bar, where ^*∗*^*p* < 0.05 vs. T0 (Holm-Sidak adjusted Wilcoxon signed rank test).

Finally, the treatment also showed a positive effect on skin plastoelasticity, with all the values obtained with torsiometry being positive at both the T1 and T2 time points. The immediate elastic recovery parameter, Ur, also reached a statistically significant improvement at T2 (Figure 7).

### 3.3. Subjects' Self-Evaluation

At the end of the trial, all the subjects participating in the study were asked to complete a questionnaire in which they had to report their satisfaction in terms of efficacy and tolerability. They had to use a scale of satisfaction ranging from absent to light, medium, marked, and very marked.  Table 1 reports the percentage of subjects reporting improvement in the different parameters. The results of the survey are very clear, as the vast majority (from 87 to 96%) of the subjects had a positive judgement.

In terms of tolerability, roughly half of the subjects reported only light bruises at the site of injection. These minor effects, however, totally disappeared in a few days. This effect was expected and thought to be due to the injection procedure itself, and in fact, the investigator judged the tolerance to the product as good/excellent in all subjects. This was also confirmed by the self-assessment, where 83% of the subjects reported excellent tolerability, and the remaining subjects reported good tolerability.

## 4. Discussion

Aesthetic treatments for skin rejuvenation are increasingly requested. This is particularly true for skin areas exposed to the other people we associate with. The general population life expectancy, at least in developed countries, is increasing, and with age, normal skin becomes rougher and loose [24–27]. The result is the formation of wrinkles, for which many people desire treatment. The use of local treatments to reduce wrinkles, skin laxity, and roughness is now rather common, and HA is one of the most widely used agents for this purpose [8, 11, 13, 18, 28–30]. Here, we have shown that a particular formulation of HA, containing hybrid cooperative complexes of high- and low-molecular-weight HA, is efficacious in reducing skin laxity and wrinkles in the neck. The positive results obtained here have been generated not only through a clinical evaluation but are also supported by different instrumental evaluations and by the subjects' judgement. Overall, the application of two injections of the studied product at a distance of 30 days resulted in a clear improvement of the neck skin, which was already evident after the first injection and was maintained, or even increased, three months after the last injection. Importantly, confirming what has already been shown for other applications [7, 9, 15, 18], positive efficacy results have been obtained with very good tolerability. It is worth noting that all the different instrumental evaluations produced concordant results with statistically significant changes in all the parameters. This is an important point, considering that instrumental evaluations have a more reliable, reproducible, and unbiased value compared to clinical evaluation.

The evidence that the antiwrinkle effect increased with time may suggest that the injections are able to induce a stimulatory effect on the dermis and epidermis, promoting new collagen formation and hence restoring tonicity and consistency to the neck skin. This hypothesis is corroborated by in vitro evidence that there is indeed biostimulation induced by HA [31].

The use of a standardized method to inject the product in a reproducible and safe way through 10 microinjections is another addition to the study in terms of data consistency among the different subjects.

The very good tolerability of the studied product makes it suitable, in addition to its activity as a single agent, for multimodal approaches to treat skin defects, an approach that has been shown to produce satisfactory results [2, 3, 6, 32].

We are aware that one limitation of the study is the relatively small number of subjects. However, the sample size used here was based on previously published studies showing the efficacy of these kinds of treatments [33].

In conclusion, only two injections of the studied product are able to induce a clear amelioration of neck skin appearance and tonicity.

## Figures and Tables

**Table 1:** Self-evaluation of the efficacy of the treatment.

	Sum of medium, marked and very marked judgements
Improvement in skin roughness	87%
Improvement in skin laxity	92%
Silhouette remodelled and more defined	91%
Lifting effect	96%
Improvement in skin suppleness	96%
Improvement in skin smoothness	91%
Improvement in skin hydration	96%

## Data Availability

The deidentified data of the Clinical Study Report (CSR) are made available upon request to the corresponding author for five years.
